# Are Hypodermic Injections of Morphine Lawful?

**Published:** 1893-02

**Authors:** 


ARE HYPODERMIC INJECTIONS OF MORPHINE LAWFUL?—A suit has just been begun in South Dakota, by a Mrs. C. M. Sweitzer, against a doctor living in Aberdeen. She alleges that the doctor repeatedly "pumped morphine into her husband, so that he became a morphine fiend," and being intellectually and morally a wreck, was unable to provide for her support. The medico-legal questions involved in this suit are of considerable interest, as what is called the morphine habit is becoming one of the notable medical signs of the day. The facility with which hypodermic injections can be made, and their possible results when long continued, make it the duty of the physician to be careful when he adopts such a line of treatment.

Some of the newspapers are clamoring for a law placing some restrictions upon the administration of morphine, alleging that it is as dangerous as the sale of liquors to minors. Whether the consent of parents or husbands is necessary to make a hypodermic injection of morphine proper, is a question which has never been before a court for decision, but if heavy doses were given and continued for a considerable length of time, without informing the patient of the character of the treatment, and evil results followed, it is very probable the physician would be liable in damages.—*Buffalo Journal.*

